# International trade and investment law: a new framework for public health and the common good

**DOI:** 10.1186/s12889-018-5486-6

**Published:** 2018-05-08

**Authors:** Louise Delany, Louise Signal, George Thomson

**Affiliations:** University of Otago, Box 7343, Wellington, New Zealand

**Keywords:** International law, Trade, Investment, Planetary health, Treaties

## Abstract

**Background:**

International trade and investment agreements can have positive outcomes, but also have negative consequences that affect global health and influence fundamental health determinants: poverty, inequality and the environment. This article proposes principles and strategies for designing future international law to attain health and common good objectives.

**Argument:**

Basic principles are needed for international trade and investment agreements that are consistent with the common good, public health, and human rights. These principles should reflect the importance of reducing inequalities, along with social and environmental sustainability. Economic growth should be recognised as a means to common good objectives, rather than an end in itself. Our favoured approach is both radical and comprehensive: we describe what this approach would include and outline the strategies for its implementation, the processes and capacity building necessary for its achievement, and related governance and corporate issues.

The comprehensive approach includes significant changes to current models for trade and investment agreements, in particular (i) health, social and environmental objectives would be recognised as legitimate in their own right and implemented accordingly; (ii) changes to dispute-resolution processes, both state-to-state and investor-state; (iii) greater deference to international legal frameworks for health, environmental protection, and human rights; (iv) greater coherence across the international law framework; (v) limitations on investor privileges, and (vi) enforceable corporate responsibilities for contributing to health, environmental, human rights and other common good objectives. We also identify some limited changes that could be considered as an alternative to the proposed comprehensive approach.

Future research is needed to develop a range of model treaties, and on the means by which such treaties and reforms might be achieved. Such research would focus also on complementary institutional reforms relevant to the United Nations and other international agencies. Advocacy by a range of communities is needed for effective change. Reform will require informed debate, determined engagement with decision-makers and stakeholders, and some agreement across health, social and environmental sectors on alternatives.

**Conclusions:**

Current frameworks of international law that govern trade and economic development need radical change, in relation to treaty processes, content, and contexts, to better attain public health objectives.

## Background

International trade and investment agreements (TIAs) affect global health, equity and justice – the common good. TIAs can have positive benefits but, from a range of health and social good perspectives, also have negative outcomes. This article focuses on ideas for *change* to such agreements and their institutional contexts, with the aim of achieving public health objectives. Because of this future focus, material in the following sections provides only a brief summary of why change is needed, and does not duplicate the growing literature on the problems for public health arising from TIAs.

For this article, we define TIAs as those agreements relevant to trade, international investment, and international intellectual property law. We define the common good as those benefits that can be shared by all, ‘that promote the full flourishing of everyone in the community. … includes, but is not limited to, public goods’[1] p.161.

International trade law governs trade in products and services; investment law covers assets; and intellectual property law has rules on what kinds of intellectual property can be protected and for how long. There is an array of TIAs in these areas, with multilateral agreements administered by the World Trade Organization (WTO), and other bilateral and regional agreements outside the WTO framework (often referred to in broad terms as free trade agreements – FTAs).

Several interrelated trends have intensified the significance of TIAs and hence their health implications. Accelerated globalisation, involving developments in transportation, technology and communication, has resulted in the extended reach and complexity of global trade. The character of TIAs has become more comprehensive, with far-reaching implications for nation states. The focus has shifted from that of tariff reduction (although that remains important) to a wide range of measures affecting many aspects of products, services, and investment [2]. ‘Trade’ and ‘investment’ agreements are tending to merge as instruments of large-scale regional economic governance, [3] with the overall number of investment-related TIAs now over 3000 [4].

Current agreements in active development include the Trade in Services Agreement (TiSA) and the Regional Comprehensive Economic Partnership; in addition to the recently signed Comprehensive and Progressive Agreement for Trans-Pacific Partnership (CPTPP).

While this article sketches ways in which TIAs should evolve to recognise health and social objectives, we recognise that giving effect to such changes would require a fundamental philosophical shift in international trade and investment policy. This article offers some directions for TIA designs and processes that are intended to be useful for governments and non-government organisations when rethinking and negotiating TIAs. It does not, however, provide solutions for the wider political questions of achieving such changes.

This following sections outline issues for public health and the common good that are created by TIAs in their wider global, legal and corporate contexts. The principles and framework proposed in the Discussion are intended to help address these issues.

### Trade and investment related agreements: Problems for public health and the common good

TIAs have a range of positive and adverse outcomes. They affect up-stream determinants of health, such as poverty and inequality, in complex and much debated ways, [5–7] p.13 and can increase inequalities between and within countries: ‘the rich can get richer, and the poor poorer.’ [8] p.8. More specifically, TIAs have implications for (1) state budgets; (2) state governance; (3) achieving common good objectives; (4) developing countries; and (5) specific health concerns in relation to both communicable and noncommunicable disease (NCDs).

#### State budgets

TIAs affect state budgets as a result of tariff reductions, [9] (especially for developing countries).

#### Governance

TIAs affect the way that states make or refrain from making policies, limit policy choices, and create contexts that result in unhealthy policies [10].

#### Common good objectives

TIAs can detract in some cases from the attainment of social, health, human rights and environmental objectives. Generally trade and investment liberalization can affect ‘power relations, social policies, employment conditions and working conditions’ [5]. TIAs can affect ‘international and domestic labor markets, …job creation, wage and labor standards, and protections’ [11]. TIAs can facilitate environment-threatening effects [12, 13]. They can influence many other areas including land tenure, [14] agricultural patterns, [15, 16] cultural traditions, privatisation, provision of health services, government procurement, [17] women’s rights, and rights of indigenous peoples [18].

While WTO TIAs provide exceptions for health and common good objectives, such exceptions are often ambiguous [11]. The concept of ‘exception’ frames common good objectives as not core to the TIA, and casts the burden of proof for meeting criteria for such exceptions on common good advocates. These criteria are often difficult to satisfy, leading to uncertain outcomes [19, 20] p.12. Many non-WTO TIAs further limit governmental ability to fulfil common good related objectives by allowing investors to take action against states [11].

#### Developing countries

Developing countries are generally the most significantly affected by TIAs. While inequalities in global distribution of income and wealth have many causes, they have emerged from a ‘historical process that was pervaded by grievous wrongs’ [21]. As Pogge argues, the resulting massive poverty is perpetuated by an ‘increasingly dense and influential web of global institutional arrangements’ such as TIAs [21].

#### Disease determinants

TIAs have direct consequences for the determinants of both communicable and noncommunicable diseases (NCDs) that arise, in part, from a basic tension between the economic growth imperatives of TIAs and health objectives. TIAs facilitate communicable disease through the global dissemination of products and people; [22] issues relating to antibiotic resistance are based partly on trade; [23] and intellectual property law affects the affordability and availability of therapeutic drugs [24] p.289–95. Efforts to address NCD risk factors can be hindered by the liberalised trade regime facilitated by TIAs. Increased competition and lower prices for consumers from TIAs can be negative for health where there are harmful products, due to increased consumption [20].

In general terms, TIAs influence NCDs because they shape the environments within which consumers make ‘choices’ on such products as foods, tobacco, and alcohol (for example, food environments) [16, 20]. A 2017 review of the effects of TIAs found ‘consistent evidence’ of associations between TIAs and both ‘increased consumption of processed foods and sugar-sweetened beverages’ and ‘higher cardiovascular disease incidence’ [25]. TIAs may restrict people’s ability to access healthy foods, eg, through impacts on agricultural practices and intellectual property protections [16]. Increased consumption of products and services with adverse health consequences, facilitated by TIAs, runs counter to health measures intended to reduce that consumption [25, 26]. Health measures designed to address NCDs might include packaging and labelling requirements, for example on alcohol containers; licences, such as those for tobacco vendors; and restrictions on advertising, promotion, labelling and product content for some food products [16, 20]. Each of these measures can infringe international trade rules [26].

### The wider context of trade and investment related agreements

International trade and investment does not exist in a vacuum, nor do the treaties that set out the relevant rules. Any analysis of how trade and trade law can be re-oriented towards achieving the common good should take account of the global and institutional settings in which TIAs operate, the complexity of international legal frameworks, and the multiple players involved, particularly the corporation.

#### Global governance and processes

Cooperative action to address the issues for public health from TIAs requires effective structures and processes for global governance. Modifications to current arrangements are needed, as many of the major global organisations prioritise financial activity and economic growth, [27] while those focused on health and its determinants appear to have less power and are not coordinated [28] p.112.

Organisations such as the World Trade Organization and the World Bank appear to have emerged as stronger elements in global governance, compared to the United Nations, over the last 60 years. Such governance is increasingly complex, with many aspects and levels [29]. The World Health Organization (WHO) has not taken a major role in the formation of TIAs or their implementation (although it has had significant influence in relation to international law on access to pharmaceuticals) [30]. While WHO may be invited to contribute to WTO dispute procedures, it has no right to do so, as made clear in the WTO dispute rules [31]. These rules state that WTO may seek information from any source related to dispute settlement, and/or set up expert review panels, but these are advisory only.

The imbalance between economic and health priorities has been intensified by the trend away from multilateralism towards bilateral, regional and mega agreements (FTAs). ‘Multilateralism’ is a concept that has normative and political connotations, with some commitment to a rule-based system, principles of universality, and governance by international organisations [32–34]. In this sense, multilateral agreements differ from regional or preferential trade agreements, even where such agreements have several parties [35, 36]. These bilateral and regional agreements are ‘negotiated outside WTO auspices’, may ‘undermine developing country interests’ [24] p.299 and hence may enforce present power imbalances. As Gostin noted, ‘FTAs enable powerful countries to lock developing countries into agreements that could not be achieved during WTO negotiations’. This may, for instance, include stronger intellectual property protections than those in the equivalent WTO agreements [24] p.299.

In both WTO TIAs and non-WTO TIAs, the present processes of treaty development can inhibit the recognition of health and common good perspectives. This can present barriers even for developed countries in achieving desired outcomes, but the barriers are more acute for developing countries with small economies. There are a number of factors affecting smaller economies as they participate in TIAs, including limited human resources and technical capacity [8] p.22–23. There are also issues relating to procedural justice in the functioning of the WTO, such as the basic bargaining process itself, which is built on political and economic power [8] p.27 [37]. Current TIA dispute resolution mechanisms have particular problems for poorer countries [37]. The mechanisms require significant resources, including legal assistance, to have any chance of success. This has ‘important implications for the justice of the overall system’ [8] p.201.

The lack of transparent, open and consultative processes has been noted by a range of official bodies, including a 2015 Australian Senate inquiry, [38] the European Union, [39] and the UN Conference on Trade and Development [40]. Concerns include the secrecy and lack of public participation opportunities in investor-state dispute settlement procedures (some newer TIAs have started to address these problems) [40].

States can be locked into treaties for long periods without sufficient review or renegotiation ability. While TIAs generally have review clauses, these need to be used effectively for change [41].

#### International legal frameworks

The multiplicity of current international legal frameworks means that international law is extremely fragmented, with little clarity around the relationships between various frameworks, and often conflicting norms [42]. As Carozza states: ‘the systemic *incoherence* of international law … is widely perceived to be an acute problem, generating many analyses of the fragmentation of international law’ [42]. A result of this fragmentation and ‘systemic incoherence’ is that international agreements do not satisfactorily set out what should happen where conflicting provisions exist in different frameworks. This is despite the interpretation provisions set out in the Vienna Convention on the Law of Treaties [43]. While the Convention is of general relevance, [44] provisions such as Article 31(3)(c) of the Convention are very high level and offer little specific guidance on resolving differences between treaties. In practice, ‘trade treaties are almost always more enforceable than treaties relating to health, human rights, labour, and the environment’ [45].

#### Corporate issues

Multinational corporations are key players in international and national policies [46]. They influence TIAs as major drivers of their architecture and benefit from them. ‘Large corporations ... hold disproportionate power in such agreements, and are the beneficiaries of their rules, which they are able to enforce through new dispute settlement mechanisms’ [47]. Multinationals influence the content of TIAs (eg, through access to draft treaty texts and lobbying power) and non-WTO TIAs strengthen the power of multinational corporations through the availability of investor-state litigation [48].

The primary duty of corporations is not to the communities within which they operate, or the broad range of common good objectives, but to their shareholders. The legal nature of the corporation means a degree of inviolability due to its limited liability [46]. Often there are differences between corporate aims as facilitated by TIAs, and health objectives, with negative implications for public health [49–53]. Attempts to ensure effective control of corporations, so as to ensure some consistency between their behaviour and human rights (including health, social and economic rights) have had, to date, very limited results. This lack of control is indicated by the work on the proposed UN Convention on Business and Human Rights [54]. Generally, TIAs have given enforceable rights to corporations, without requiring enforceable obligations from them.

## Discussion

The above sections set out specific, systemic, and institutional problems that TIAs, in their global governance contexts, pose for public health and other common good objectives. How can TIAs be redesigned to better align with health objectives?

A number of studies have suggested ways in which some of the negative implications of TIAs could be addressed (eg, [55–59]). In particular, UNCTAD has provided guidelines for reforming the making and implementation of investment treaties [60]. Their ‘Investment Policy Framework’ suggests ways to protect states’ rights to regulate, and to improve dispute processes [61]. Smith et al. provide a detailed agenda for action [62].

This article builds on these and other suggestions to propose a systematic and comprehensive approach to transform TIAs for the common good. It incorporates general principles, a framework for new law, and the identification of institutional, structural and procedural features that would be necessary to support that framework. The approach we suggest would not reject the importance of international trade and the rules for its regulation, but would promote a different and evolved type of TIA that would more effectively recognise multiple common good perspectives.

### Suggested principles for trade and investment related treaties

These suggested principles, as well as the following framework, respond to the issues posed by TIAs as outlined in the Background. They would be used as benchmarks to underpin common good perspectives for future TIAs by relevant policy communities, treaty drafters and public health advocates.

The main purpose of the principles is the provision of a succinct ‘checklist’ for evaluating, from a health and common good perspective, the desirability of a particular proposed or current treaty. The principles would help guide decisions on whether to enter into or renew TIAs, of which kind, and in accordance with which processes. The principles would help frame questions, and indicate answers, on the likely effects of specific strategies and provisions embodied in treaty text, to be used perhaps in impact assessments.

A second purpose of the suggested principles is that they could, in some cases, offer ideas for inclusion in introductory treaty text (eg, preambular or purpose provisions). Such inclusion might function as a guide to interpretation, and might also be useful in providing links to other instruments of international law.

The principles below are divided into those: (a) relevant to the processes by which treaties are developed; and (b) those relating to the objectives of the treaties themselves. Further details on how the principles might be operationalised are given later in the article.

#### Principles for TIA development processes

The essential bases for just and sustainable treaty processes include:

1. Support for the effective participation of countries and the participation of citizens within these countries in developing policies for TIAs, with special emphasis given to poorer countries and population groups;
2. Support for multilateralism; that is, an approach that involves a commitment to rule-based principles, and norms of openness and universality, preferably within global governance structures, processes and institutions, rather than regionally based treaties;
3. Transparent, fair, open and consultative processes for TIA development and implementation;
4. Recognition, throughout the treaty development process, of the need for provision for, and effective use of, review clauses in TIAs.

#### Principles for TIA objectives

5. Explicit incorporation of social, health, human rights, and environmental objectives as core to TIAs, and explicit support for the UN Sustainable Development Goals (SDGs), especially those relevant to:

- eliminating poverty
- reducing inequalities and redressing power imbalances
- protecting and advancing planetary health (‘the health of human civilisation and its underpinning natural systems’ [63]);

6. Recognition of economic growth as a means to common good objectives, rather than an end in itself; through for example explicit reference to relevant human rights law, in particular the International Covenant on Economic, Social and Cultural Rights; [64]
7. Effective control of international corporations by the international community through international law;
8. Redressing the specific situations of historically harmed states; [65] for instance through the provision for special and differentiated treatment for least developed countries. Such ‘special treatment’ could refer not only to orthodox issues such as tariffs, but also specific forms of exceptions and carve outs, and by emphasis on freedom of governmental regulation;
9. Support for human rights principles, incorporating a collective vision of human rights. That is, explicit recognition that the concept of ‘human rights’ should go beyond traditional individualistic connotations, and envision a ‘collective right to public health – a right applied at the societal level to address underlying determinants of health’ [66].

### A framework for future treaties

We favour a comprehensive approach to designing new TIAs. This responds to the need to implement the SDGs; and to the requirement for fundamental change to promote global health and equity in ways that do not compromise environmental sustainability [63]. The comprehensive, or ‘planetary’, approach that we favour for TIA design, based on the principles outlined above, would integrate health, environmental and social objectives. Such integration would be the cornerstone of redesigned TIAs that are fit for the twenty-first century.

The comprehensive approach towards redesigning a new framework for TIAs would be buttressed by improved processes for treaty development, enabled in turn by strengthened institutional capacity. New TIA frameworks would require, for effective implementation, governance support from relevant international institutions and attention to issues relating to corporate behaviour.

The following part of our article is structured in eight sections. The first five are: (1) outline of comprehensive approach integrating a range of objectives, (2) strategies to give effect to this approach, (3) necessary processes, (4) capacity building, and (5) governance and corporate issues. Section (6) touches on connections between international law and national law; followed by section (7) which pulls together ideas on enforceability; and then section (8) which briefly notes a more limited approach to redesigning TIAs.

### Section 1: Comprehensive approach – A set of common good goals

TIAs would provide for health, environmental and other common good perspectives to be explicitly recognised as core treaty objectives along with economic goals. Treaty chapters would set out how these core objectives would be achieved. Under current TIA frameworks, national measures relating to goals such as environmental protection and public health – while often referred to in preambular treaty statements – are in practice generally permissible only as exceptions. These measures are often difficult to justify in accordance with the current wording of exceptions and their interpretation [19] ch.8. Reframing such measures as legitimate treaty objectives in their own right, for example, along the lines proposed for environmental objectives by the International Institute for Sustainable Development *Model International Agreement on Investment for Sustainable Development*, [67] would help address these difficulties.

### Section 2: Strategies to give effect to the comprehensive approach

Strategies to give effect to a set of integrated social and environmental objectives would include some of those proposed by the United Nations Conference on Trade and Development (UNCTAD) [68], prioritising the needs of developing countries [69]. Such strategies would cover issues relating to dispute resolution, exceptions, regulatory responsibility and deference to specified health, environmental and human rights agreements.

#### Dispute resolution

There are two main international methods of TIA dispute resolution. One is that provided in WTO treaties, where only nation states may take action in relation to other states. The other is the method in most non-WTO treaties. This, as well as state-to-state action, allows *investors* to take action against states through investor-state dispute settlement (ISDS).

Modifications to non-WTO treaties would include either significant change to ISDS or its removal. Concerns about ISDS include process issues, and ‘chilling’ effects due to both process and outcomes [70]. ‘Chilling’ is government inaction because of potential or real threats of legal action. Modifications to ISDS could involve provision for appeals; a more ‘judicial’ approach to appointing impartial dispute decision makers; greater transparency of dispute decision-making; the ability for the public, NGOs, and relevant sectors to have input to hearings; and published decisions with precedent value [68] p.147–8.

Some countries have demonstrated that ISDS is not a necessary element of TIAs: one example being the ‘Brazilian’ model which has trade facilitation agreements without ISDS [71]. An approach that significantly modifies traditional ISDS is included in the Comprehensive Economic and Trade Agreement (CETA) between the EU and Canada. This is intended to create, for disputes arising under CETA, an Investment Court System including a permanent Appellate Tribunal, with ethical rules such as preventing conflicts of interest (see Articles 8.27 to 8.30) [72]. This aspect of CETA is not, however, yet in force and is subject to ratification procedures.

The removal of ISDS, rather than its modification, would necessitate reliance on domestic court systems in the country hosting the investment, or some form of state-to-state dispute settlement, [73] or new mechanisms such as that envisaged for CETA. Other alternatives include a World Investment Court or, as proposed by the OECD, a Multilateral Investment Court [74–76]. Domestic court solutions are emerging in treaties entered into by South Africa, Brazil and other countries [77].

Current state-to-state dispute resolution processes, whether those in WTO or non-WTO treaties, may also inadequately reflect health perspectives. Members of dispute resolution bodies tend to have backgrounds in trade and trade law, [78] and have world views to which health objectives are not central. While input from other disciplines and sectors is permissible, and often sought, there are no requirements in WTO processes to ensure that input from other sectors is obtained and taken into account. Refinements to WTO processes, and state-to-state dispute procedures in non-WTO treaties, could ensure that panellists or Appellate Board members, as well as those involved in state-to-state disputes under FTAs, have an appropriate environmental or health background in relevant cases [79].

#### Exceptions and ‘carve outs’

To complement changes to dispute resolution systems, and to give effect to a set of integrated objectives, changes would also be needed in TIA provisions that relate to ‘exceptions’ and ‘carve outs’ for health, social and environmental goals. Ideally, ‘exceptions’ and ‘carve outs’ for such goals would not be needed, given that new TIAs would give equal weight to health, social and environmental goals, along with those relevant to economic growth. However, given that some forms of ‘exceptions’ may continue, we propose that (1) the use of carve outs be expanded to put beyond doubt the exclusion of treaty provisions for some products and services (eg, tobacco, pornography); and (2) the concept of ‘exception’ be replaced by provisions relating to prioritisations of treaty goals, with mechanisms to ascribe priorities between different objectives, for example:

i. Explicit priority for some specified objectives. At one extreme, health or environmental aims would not require justification and hence would be ‘self-judged’ by the country concerned (following the example of exceptions on security grounds);[80]^Article 10.4^
ii. Criteria such as significant mortality and morbidity potential, as well as international targets for reducing relevant mortality and morbidity, as a basis for recognising the primacy of measures conducive to human, animal or plant life and health.

#### Affirming governmental regulatory responsibility and limiting investor privileges

Current treaties include a range of provisions that are aimed at bolstering the ‘rights’ of investors, effectively reducing the scope for government regulation. Three major and often inter-related examples are provisions for ‘fair and equitable treatment’; ‘expropriation’; and those relating to intellectual property.

‘Fair and equitable treatment’ (FET) provisions often include commitments to fulfil ‘legitimate expectations’ for investors. FET is an important ground for litigation that is often successful for investors, and can be a major contributor to the ‘regulatory chill’ factor in investment matters. Agreements should exclude commitments to fulfil ‘legitimate expectations’ for investors, or define such expectations more narrowly. This approach has been proposed by UNCTAD, noting a range of options in this area [81] (p 104–114). One example of a model treaty which arguably accords with one of the UNCTAD options is Canada’s model agreement for the promotion and protection of investments, see Article 6(2) [80]. The Canada-EU CETA also qualifies what is meant by ‘fair and equitable treatment’, and limits the concept of ‘legitimate expectations’ to situations where a specific promise or representation is made by the State (Article 8.10) [72]. We consider it may be simpler to exclude provisions relating to ‘fair and equitable treatment’ (as well as ‘legitimate expectations’) altogether from TIAs.

Investment chapters in current TIAs usually have specific sections on expropriation. The chapters can be linked with issues relevant to ‘fair and equitable treatment’ as well as legitimate expectations. While such provisions acknowledge that governments may take regulatory action, even when negative effects on investment may be expected, it is often difficult to distinguish such measures from those which are considered to be indirect expropriation and hence liable to compensation [82].

We propose that: (1) no governmental action taken in relation to an issue specified in provisions covered in carve outs may be considered as forms of expropriation, direct or indirect; and (2) no governmental action taken in relation to an issue that is prioritised in accordance with the mechanisms proposed above (for ascribing priorities between different objectives) could be considered as any form of expropriation. Alternatively, TIAs could simply exclude altogether the concept of indirect expropriation and focus on explicit definitions of direct appropriation.

Recent treaty development in non-WTO law has seen increased protections for the holders of intellectual property, and often include intellectual property in the definition of investment [83, 84]. TIAs that are redesigned for health should limit intellectual property protection for investors.

#### Deference to other instruments of international law

Redesigned treaties that integrate common good objectives should include provisions to clarify the relationship between TIAs vis-à-vis international health and environmental law. TIAs should refer and defer to other specified international health, environmental and human rights agreements, [85] eg, the United Nations Convention on the Rights of the Child, and the Framework Convention on Tobacco Control.

### Section 3: Treaty development processes

Treaty law does not come into the world of its own accord. The processes by which TIAs are developed should be improved to ensure that common good objectives are fully recognised and to ensure that the interests of the least advantaged are protected.

Improvements would include:

- Transparent negotiation processes, greater consultation, participation, and openness to public and legislator scrutiny – eg, through critical points where draft treaty texts are published, as was done by the EU for the Transatlantic Trade and Investment Partnership [86]. This is, according to the European Commission, to be the rule for all their trade negotiations, as part of active engagement ‘with civil society and the public at large in the context of the civil society dialogues and citizens’ dialogues’ [39] p.19. Engagement would include public policy papers developed and published at early stages of treaty development, with broad negotiating positions stated (as in the EU) [86] pp.7–8. While such transparency is essential, it does not itself enable true consultation and participation. This would also require representatives of civil society being engaged in development of draft treaty provisions, negotiation, monitoring of any agreements once implemented and evaluation.
- Open development of national treaty positions, perhaps through developing ‘model’ treaty templates, as is done by many countries of the Organization for Economic Co-operation and Development, [87] p.144 and other states. Examples include the Model Text for the Indian Bilateral Investment Treaty and those developed by regional groups such as the Southern African Development Community Model Bilateral Investment Treaty.
- The use during the development of treaty positions of impact assessments that capture a wider range of effects, using tools such as Health Impact Assessments and the UN Global Policy Model [88]. These would identify, during the negotiations, the advantages and disadvantages that may arise from a specific TIA for different groups in society, along with the potential impacts for health and other common good issues. Treaty audit tools, such as national interest analyses, or health impact assessments, [89] could identify those groups in society who may suffer disadvantage as a result of a particular TIA (eg, loss of jobs, reduction of wages, increased prices of some products). Such analyses could also identify health outcomes which could be adversely affected (eg, with reference to health services) [90].

### Section 4: Capacity-building and institutional support for treaty development and implementation

Treaty development and implementation requires attention to the many historical and institutional factors that negatively affect achieving health and environmental objectives. This is relevant to both developed and developing countries, but particularly recognises the disadvantages facing developing countries. Most critically, the health, environmental and human rights sectors need an effective place at the TIA negotiating table.

The health community needs greater capacity at all levels - international, regional and state - to enable effective engagement with trade policy and issues relating to TIAs. Health officials in both developing and developed countries need skill development, both in treaty negotiation and in understanding the implications of international law (existing and proposed) for their domestic law. At the international level, the WHO has limited legal in-house capacity, and does not appear to be able to support developing countries to the extent required. Ensuring technical expertise in negotiating and implementing TIAs (especially for developing countries) [91] should be seen as a required role for both WHO and the WTO.

Improvements would therefore include:

- Ensuring greater capacity for health, environmental and human rights engagement in TIA development;
- Mandating specific public health, environmental and human rights expertise in WTO technical capacity;
- International health funding to support training and capacity building for developing countries in trade and health policy.

### Section 5: Global governance and structural issues

#### Changes in international contexts

Coherent and effective global governance is needed to support health equity, environmental sustainability, and the implementation of the SDGs. At the most general level, effective global governance for health purposes may require the reform of WHO in order to provide it with greater powers and structures that are more effective [92]. United Nations (UN) mechanisms are needed to address cross-sector areas of activity. Decisions are also needed on where the UN should provide a global focus for action (following the model of UN action on both HIV/AIDS and NCDs) [93, 94]. Attention to global governance structures would also include the reinvigoration of multilateralism (structures, processes, institutions and law), given the potential for non-multilateral approaches to reinforce present power imbalances.

Coherence in the general international law framework is needed. Appropriate hierarchies in international frameworks should be supported, for example, through interpretation invoking the Vienna Convention; [44] with priority to be given international human rights law vis-à-vis other instruments of international law. New international health law is needed (eg, on alcohol, non-nutritious food). On a more general scale, the proposed Global Health Convention is one model for new law [95].

#### Changes relevant to corporations

The successful implementation of new TIAs that would meet health, environmental, human rights and other common good objectives requires attention to corporate structures and behaviour. This is because, along with states, corporations are the main actors in TIAs.

Addressing corporate issues at international and national levels would involve, amongst other things:

- An international convention to build on the Ruggie concepts of business responsibilities in relation to human rights, as resolved by the United Nations Human Rights Council in 2014 [96]. This convention would have binding force, as proposed by 2017 discussions on a new convention; [54, 97] and should establish primacy of human rights over TIAs [54 85]. The elements of the October 2017 draft of the new convention include a number of valuable proposals, including the proposed duty of state parties to prepare human rights impact assessments prior to the conclusion of trade and investment agreements (Principles1.2) and some provisions for enforcement; [54]
- Incentives in TIAs for corporate compliance with human rights, health, environmental and other common good objectives, and effective sanctions on corporations for non-compliance;
- Action by nation states applicable in their own jurisdictions, for example provision for investor responsibility in state legislation, with conditions attached to corporate investment by foreign investors, and processes to enable individuals and groups to take effective action against corporations.

### Section 6: Connections between international and national levels

In accordance with any disadvantages identified by national interest analyses or health impact assessments, nation states should, through their domestic law, counter the identified adverse effects from TIAs as much as possible. Such law would need to come into effect at the same time as the treaty. For example, compensatory taxation measures for disadvantaged groups (such as those whose employment is affected) may be appropriate. National law should require contracts for foreign direct investment to explicitly recognise the responsibility of national governments to regulate for the common good. Foreign direct investment should also be conditional on compliance with stated health or environmental goals, for example, nutritional goals; and national law should stipulate that any breach of such conditions would invalidate any possibility of investor-state litigation.

### Section 7: Enforceability

Many concepts proposed in this article are intended in general terms to promote the enforceability of common good objectives vis-à-vis the economic growth objectives of TIAs and the financial interests of corporations. Mechanisms to ensure such enforceability should occur at several levels: through a range of strategies within TIAs; within other legal instruments relating to health and environmental objectives (for example the FCTC and the Paris Agreement); through any convention on the responsibilities of businesses in relation to human rights; and within national legislation. While ideally these mechanisms should interlink, they may also be implemented independently.

In summary, enforceability mechanisms could include:

#### TIA level

Assertion in treaty text of the equal weight to be given to common good objectives, and the primacy of human rights law, over pecuniary interests would render it more difficult to initiate dispute procedures involving challenges to common good objectives. Treaty text would further provide that non-compliance with common good objectives would render invalid any possible dispute involving investor rights (under state-to-state rules or ISDS), and render states and investors ineligible for benefits under the relevant TIA;

#### Other international agreements

Instruments such as the Paris Agreement should specifically provide for the supremacy of climate control objectives versus those relating to economic growth;

#### Overarching international law

An international convention on business responsibilities in relation to human rights would have its own implementation and enforcement mechanisms. These would include provisions for states to enact disclosure requirements, contractual preferences for corporations with appropriate histories of human rights and environmental compliance; and imposition of criminal liability for breaches of human rights, administrative and monetary sanctions;

#### National legislation

Many of the ‘elements’ proposed for a convention on business responsibilities and human rights need not await finalisation of such a convention – likely, at best, to be many years away. Hence, national legislation could enact a general framework with which any new TIA entered into by the relevant state would need to comply. Such a framework could include, perhaps, a ‘model’ TIA appropriate to that country. The framework would provide that draft TIAs would (i) need human rights impact assessments; (ii) enable conditions to be attached to corporate investment; (iii) allow individuals and groups to take effective action against corporations; (iv) require contracts for foreign direct investment to explicitly recognise the responsibility of national governments to regulate for the common good, ensuring compliance with stated health or environmental goals; and (v) stipulate that any breach of such conditions would invalidate investor-state litigation and state-to-state dispute resolution procedures.

### Section 8: Limited approaches to redesign of TIAs

A limited approach to redesigning TIAs could be adopted in the short term, instead of the proposed comprehensive framework. This limited approach could consist of integrated objectives, strategies to achieve them, and improved processes and capacity building. Individual features that could be implemented on their own might include:

- Improvements to dispute resolution – eg, abolition of or modification to ISDS
- Reductions in investor protections (eg, those relevant to ‘legitimate expectations’) and rolling back recent increases in investor intellectual property protections
- Some deference to other instruments of international law
- Greater technical capacity and skill building for health sector involvement in TIAs

A broadened scope for ‘exceptions’ or ‘carve outs’ could ensure less dependence on the present strict ‘necessity’ tests. Revised phrasing for exceptions could recognise that distinctions (such as the use of sustainable production methods) are legitimate for environmental protection.

This more limited approach, based on the adoption of one of more such elements, might address some issues posed by TIAs, but would not succeed in creating the fundamental framework change that we consider is needed.

## Conclusions

Current TIAs have multiple adverse implications for health and the common good. Rethinking current frameworks of international law governing trade and economic development is possible, and is necessary to attain public health objectives and help give effect to the SDGs. We have put forward ideas for a comprehensive approach to the redesign of international trade law, consisting of integrated objectives, strategies to achieve them, and improved processes and capacity building. Such redesign would be facilitated by significant changes to global governance; and would address specific issues posed by corporate structures and practices. The redesign of TIAs, and improvements in general international and corporate contexts, should also be mirrored and given effect in national law.

Some of these ideas would be susceptible to a ‘mix and match’ or incremental approach. Nevertheless, we strongly favour a re-envisioning of basic frameworks, as implemented by the comprehensive approach outlined.

We conclude that ‘model’ treaties are needed to demonstrate how new TIA law could look in practice, as well as ideas on how new law might be realised. Research is needed to develop details of the range of models and the institutional underpinnings to ensure their efficacy.

International law that is health promoting, sustainable, and supportive of the SDGs, will require advocacy for real change. Change will require informed debate, determined engagement with decision-makers and other stakeholders, some agreement on alternatives, and the development of alliances between health communities and other groups interested in the common good [98–100].

## Abbreviations

CETA: Comprehensive Economic and Trade Agreement
FET: Fair and equitable treatment
FTA: Free trade agreement
HIA: Health Impact Assessments
ISDS: Investor-state dispute settlement
SDGs: Sustainable Development Goals
TIA: Trade and investment agreement
UN: United Nations
UNCTAD: United Nations Conference on Trade and Development
WHO: World Health Organization
WTO: World Trade Organization
